# Current understanding of genetic associations with delayed hypersensitivity reactions induced by antibiotics and anti-osteoporotic drugs

**DOI:** 10.3389/fphar.2023.1183491

**Published:** 2023-04-26

**Authors:** Chih-Hsuan Wung, Chuang-Wei Wang, Kuo-Chu Lai, Chun-Bing Chen, Wei-Ti Chen, Shuen-Iu Hung, Wen-Hung Chung, Taiwan Severe Cutaneous Adverse Reaction Consortium

**Affiliations:** ^1^ Chang Gung Memorial Hospital, Taoyuan, Taiwan; ^2^ Department of Dermatology, Drug Hypersensitivity Clinical and Research Center, Chang Gung Memorial Hospital, Taipei and Keelung, Taiwan; ^3^ Cancer Vaccine and Immune Cell Therapy Core Laboratory, Department of Medical Research, Chang Gung Memorial Hospital, Taoyuan, Taiwan; ^4^ Chang Gung Immunology Consortium, Chang Gung Memorial Hospital and Chang Gung University, Taoyuan, Taiwan; ^5^ Department of Dermatology, Xiamen Chang Gung Hospital, Xiamen, China; ^6^ Department of Physiology and Pharmacology, College of Medicine, Chang Gung University, Taoyuan, Taiwan; ^7^ Graduate Institute of Biomedical Sciences, College of Medicine, Chang Gung University, Taoyuan, Taiwan; ^8^ Division of Hematology and Oncology, Department of Internal Medicine, New Taipei Municipal TuCheng Hospital (Built and Operated by Chang Gung Medical Foundation), New Taipei City, Taiwan; ^9^ College of Medicine, Chang Gung University, Taoyuan, Taiwan; ^10^ Whole-Genome Research Core Laboratory of Human Diseases, Chang Gung Memorial Hospital, Keelung, Taiwan; ^11^ Immune-Oncology Center of Excellence, Chang Gung Memorial Hospital, Linkou, Taiwan; ^12^ Graduate Institute of Clinical Medical Sciences, College of Medicine, Chang Gung University, Taoyuan, Taiwan; ^13^ Institute of Pharmacology, School of Medicine, National Yang Ming Chiao Tung University, Taipei, Taiwan; ^14^ Department of Dermatology, Beijing Tsinghua Chang Gung Hospital, School of Clinical Medicine, Tsinghua University, Beijing, China; ^15^ Department of Dermatology, Ruijin Hospital, School of Medicine, Shanghai Jiao Tong University, Shanghai, China; ^16^ Genomic Medicine Core Laboratory, Chang Gung Memorial Hospital, Linkou, Taiwan

**Keywords:** delayed hypersensitivity reactions, human leukocyte antigens, Stevens-Johnson syndrome, toxic epidermal necrosis, drug reactions with eosinophilia and systemic symptoms

## Abstract

Drug-induced delayed hypersensitivity reactions (DHRs) is still a clinical and healthcare burden in every country. Increasing reports of DHRs have caught our attention to explore the genetic relationship, especially life-threatening severe cutaneous adverse drug reactions (SCARs), including acute generalized exanthematous pustulosis (AGEP), drug reactions with eosinophilia and systemic symptoms (DRESS), Stevens–Johnson syndrome (SJS), and toxic epidermal necrolysis (TEN). In recent years, many studies have investigated the immune mechanism and genetic markers of DHRs. Besides, several studies have stated the associations between antibiotics-as well as anti-osteoporotic drugs (AOD)-induced SCARs and specific human leukocyte antigens (HLA) alleles. Strong associations between drugs and HLA alleles such as co-trimoxazole-induced DRESS and HLA-B*13:01 (Odds ratio (OR) = 45), dapsone-DRESS and HLA-B*13:01 (OR = 122.1), vancomycin-DRESS and HLA-A*32:01 (OR = 403), clindamycin-DHRs and HLA-B*15:27 (OR = 55.6), and strontium ranelate (SR)-SJS/TEN and HLA-A*33:03 (OR = 25.97) are listed. We summarized the immune mechanism of SCARs, update the latest knowledge of pharmacogenomics of antibiotics- and AOD-induced SCARs, and indicate the potential clinical use of these genetic markers for SCARs prevention in this mini review article.

## 1 Introduction

Adverse drug reactions (ADRs) are one of the general causes of death worldwide (Shoshi et al., 2015). In America, ADRs represented the fourth leading cause of death (Insani et al., 2021). 10%–15% ADRs contribute to type B reactions which are bizarre and unexpected reaction (Wilkerson and Drug Hypersensitivity Reactions, 2022). Type B ADRs, predominantly T cell-mediated drug-induced delayed hypersensitivity reactions (DHRs), presents variable severity and clinical diagnosis, from mild skin injury such as maculopapular exanthema (MPE) to life-threatening severe cutaneous adverse drug reactions (SCARs) including acute generalized exanthematous pustulosis (AGEP), drug reactions with eosinophilia and systemic symptoms (DRESS), Stevens–Johnson syndrome (SJS), and toxic epidermal necrolysis (TEN) (Copaescu et al., 2020). SCARs are rare, but they have high mortality rates (AGEP: <5%, DRESS: 5%–10%, SJS/TEN: 10%–40%) (Bjornsson and Bjornsson, 2017; Oh et al., 2021; Huang et al., 2022). In global, the incidence of SCARs is reported to be 0.4–1.2 per million per years (Verma et al., 2013). However, the incidence rates of SCARs from European and the United States show a divergence from Asian population (Tempark et al., 2022). A Germany study revealed that the incidence of SCARs was 1.53–1.89 per million people per year (Mockenhaupt, 2012), while a Philippines study reported a prevalence of 6.25 per 10,000 people from 2011-2015 (Guzman and Paliza, 2018). Moreover, the incidence of SJS and TEN in the European population is estimated to be 1–6 and 0.4–1.2 per million people per year respectively, while the incidence of them in the Korean was 3.96–5.03 and 0.94–1.45 per million people per year respectively. SCARs can be affected by racial, genetic and drug category difference, so results from various countries should be evaluated carefully and separately (Yang et al., 2016; Duong et al., 2017; Kang et al., 2021).

AGEP, DRESS, and SJS/TEN are three important phenotypes of SCARs that we will review in this article (Yang et al., 2021). Over 90% cases of AGEP are related to drugs, especially antibiotics such as aminopenicillins (Gammoudi et al., 2018), cephalosporins (Torres-Navarro et al., 2020), sulfonamides (Spadaro et al., 2021), vancomycin (Pettit et al., 2020), pristinamycin (de Sousa et al., 2018) and quinolones (Feldmeyer et al., 2016; Martinez-De la Torre et al., 2021). Although SJS/TEN are mainly associated with anti-epileptic drugs, some kind of antibiotics including penicillins, sulfonamides, and macrolides contribute to SJS/TEN (Kloypan et al., 2021; Pejčić, 2021). Various studies of DRESS induced by antibiotics are disclosed including sulfonamides (Asyraf et al., 2022), amoxicillin (Abdin et al., 2019), minocycline (Ganeshanandan and Lucas, 2021) and vancomycin (Clark et al., 2020). Apart from antibiotics-related DHRs, strontium ranelate (SR), one of anti-osteoporotic drugs (AODs), has been reported as causative agent of SCARs (Chen et al., 2021a). Osteoporosis is represented as bone fragility by a loss of bone material and deteriorating bone-micro-architecture (Wung et al., 2021). Several AODs can be used to treat osteoporosis including bisphosphonates, selective estrogen receptor modulators, senosumab, romosozumab, SR and calcitonin (Chiodini et al., 2020). The prevalence of bisphosphonates-induced cutaneous ADRs (CADRs) is relatively low, while the incidence of SR-induced SCARS is at moderate risk (Chen et al., 2021a). In light of the severity of SCARs, it is crucial to recognize the integration of T cell and pathogenesis of antibiotics- and SR-related DHRs (Mustafa et al., 2018).

## 2 Mechanism of SCAR

The pathogenesis of SCARs is strongly associated with specific human leukocyte antigens (HLA), T cell receptor (TCR), drug or its metabolites and further T cell-mediated immune response (Jantararoungtong et al., 2021; Hung et al., 2022). In human genome, HLA system is the most polymorphic genetic region, resulting in presenting a variety of peptides (Negrini and Becquemont, 2017). In addition, regional and ethnic difference also express HLA alleles variation (Barbarino et al., 2015). High polymorphic and heterogenetic properties of HLA molecules enable immune system not only with an advantage to defy diverse microorganisms and antigens the host encounters but also with a disadvantage to interact with various drugs and its metabolites (Crux and Elahi, 2017). Currently, four hypothetic models for the mechanism responsible for relationship between HLA molecule-dependent manner and T cell-mediated SCARs have been proposed: altered peptide repertoire model, hapten model, pro-hapten model and pharmacological-interaction model (Miyagawa and Asada, 2021a). It is unique that these models are non-mutually exclusive, which means a specific mechanism may be prevalent for a certain drug but not for another (Negrini and Becquemont, 2017). The association between HLA alleles and SCARs in different kind of medications has increasingly been reported in recent 2 decades (Wang et al., 2022a). Recent studies show that different kinds of drugs might display single or overlapped SCARs and molecular mechanism (Villani et al., 2017). In this paragraph, we summarize the mechanism of different kinds of SCARs.

### 2.1 Immune mechanism of AGEP

AGEP has been characterized as T cell-mediated neutrophilic inflammatory reaction (Wang et al., 2022b). After exposure to drugs, antigen presenting cells (APCs) presents the antigen with HLA molecule to cause activation of cluster of differentiation (CD) 4 and CD 8 T cells, referred to as drug-specific T cells (Szatkowski and Schwartz, 2015). During the development of AGEP, drug-specific T cells as well as cytotoxic T lymphocyte (CTL) released cytotoxic proteins including granulysin, granzyme B, and perforin play an important role (Dotiwala et al., 2016; Feldmeyer et al., 2016). Granzyme B can induce keratinocytes’ apoptosis, leading to subcorneal vesicles formation (Verneuil et al., 2011; Feldmeyer et al., 2016). There are many mediators and cytokines involved in recruitment of neutrophils (Svoboda et al., 2022). T helper 1 cells (Th1 cells) can produce predominant cytokines including granulocyte-macrophage colony-stimulating factor (GM-CSF), tumor necrosis factor-α (TNF-α), interferon-γ (IFN-γ) to enhance neutrophil recruitment (Piipponen et al., 2020; Wang et al., 2022b). Additionally, Th17 cells can directly recruit neutrophils via secretion of IL-17 and IL-22 (Pelletier et al., 2010; Klaewsongkram et al., 2021). CXC motif chemokine ligand 8 (CXCL8), also known as interleukin-8 (IL-8), is a potent neutrophil chemotactic chemokine and is responsible for pustule formation by neutrophil aggregation (Metzemaekers et al., 2020). Moreover, IL-36 receptor antagonist (IL-36 RA) deficiency in some AGEP patients resulted in reinforcing the expression of TNF-α, CXCL8, IL-1, IL-6, IL-17, and IL-23 (Feldmeyer et al., 2016; Liu et al., 2020).

### 2.2 Immune mechanism of SJS/TEN

SJS and TEN are rare but life-threating dermatologic diseases and represented as T cell-mediated keratinocyte death (Arora et al., 2021). TEN is always triggered by drugs, while SJS almost triggered by drug and small part of infection (Nowsheen et al., 2021). Both are characterized by the acute onset of blister formation at the epidermal layer, and hemorrhagic and erosive lesions at the mucous layer after drug exposure (Miyagawa and Asada, 2021b). In the early stage of SJS/TEN, blister fluid over epidermis was mainly infiltrated with CTLs, Natural killer (NK) cells and NK/T cells (Neerukonda and Stagner, 2021). Initially, CTLs cause keratinocyte apoptosis and cell-cell contact-dependent epidermal damage through perforin and granzyme B pathway (Tohyama and Hashimoto, 2012). Secondly, CTLs and NK cells secrete Fas ligand (FasL) to bind Fas on keratinocyte surface and activate Fas-FasL pathway, leading to keratinocyte apoptosis (Hasegawa and Abe, 2020). Then, granulysin, secreted by CTLs and NK cells, is a pro-apoptotic protein which demonstrates widespread cytotoxicity indirectly (Su et al., 2017; Sadek et al., 2021). In addition, IL-15 secreted by keratinocyte and CTLs themselves was thought to enhance the expression of granulysin-mediated apoptosis (Stern and Divito, 2017; Neerukonda and Stagner, 2021). In the late stage, granulysin could stimulate C-C motif chemokine ligand 20 (CCL20) expression in monocytes, leading to monocyte infiltration (Tewary et al., 2010). Monocyte could not only enhance consistent CTLs’ cytotoxicity but also activate TNF-receptor 1 (TNF-R1) mediated apoptosis pathway by TNF-α secretion, resulted in further epidermal destruction (Tohyama et al., 2012; Su et al., 2017; Kuijper et al., 2020). Besides, Olsson-Brown et al. (2022) reported a mechanism of TNF-α-induced matrix metalloproteinase 9 (MMP9) expression of keratinocytes in SJS/TEN. Recently, monocytes might play an important role in keratinocyte necroptosis, another keratinocyte’s death mechanism (Saito, 2014). Necroptotic cells cause inflammation by releasing lots of pre-inflammatory cytokine, while apoptotic cells cause cell death without inflammation (Hasegawa and Abe, 2020). Multiple and detailed mechanism involved in SJS/TEN will be gradually investigated in the future.

### 2.3 Immune mechanism of DRESS

DRESS is a rare but severe cutaneous and systemic drug-delayed hypersensitivity reaction, mediated by T cell activation (Schunkert and Divito, 2021). Drugs, drug’s metabolites and/or coincidental viral infection such as human herpesvirus (HHV)-6 and 7, cytomegalovirus (CMV) and Epstein-Barr virus (EBV)-induced T cell activation seem to play an important role in the pathogenesis of DRESS (Chen et al., 2018a). Th2 cells and CTLs induced by DRESS activate hypersensitivity response leading to skin damage and cause organ damage, respectively (Sharifzadeh et al., 2021). Th2 cells secrete IL-4, IL-5, and IL-13 to recruit macrophages, eosinophils and mast cells, resulted in inflammatory reaction (Kang et al., 2020). IL-5 is contributed to eosinophilic differentiation and expansion at the inflammatory skin site. C-C motif chemokine 8 (CCR8) + Th2 cells belong to IL-5-enriched subgroup associated with eosinophilic inflammation (Endo et al., 2014). Besides, innate lymphoid cells (ILC) can also secrete IL-5 to increase eosinophil recruitment in the damage site such as skin, target organs and even peripheral blood (Cardones, 2020). Thymus and activation-regulated chemokine (TARC/CCL17), produced by keratinocytes, not only can recruit Th2 cells into inflammatory site, but also may be associated with disease severity (Vestergaard et al., 2000; Stirton et al., 2022). Besides, pro-inflammatory cytokines including TNF-α, IFN-γ, IL-2, IL-6, IL-15 and granulysin were reported to be associated with DRESS (Weinborn et al., 2016; Wang et al., 2022b). On the other hand, several studies revealed that DRESS is associated with regulatory T (Treg) cells and Th17 cells activation (Pichler and Brüggen, 2022). Treg cells are expanded by classical monocytes secreted IL-10 in the acute stage of DRESS, while T17 cells are proliferated by pathological monocytes in the resolution stage (Shiohara and Mizukawa, 2019). T cell shift from Treg cells to Th17 cells in the subacute phase is probably derived from IL-6 secretion from pathological monocytes (Ushigome et al., 2018).

## 3 HLA susceptibility to drug-induced SCAR

**FIGURE 1:**
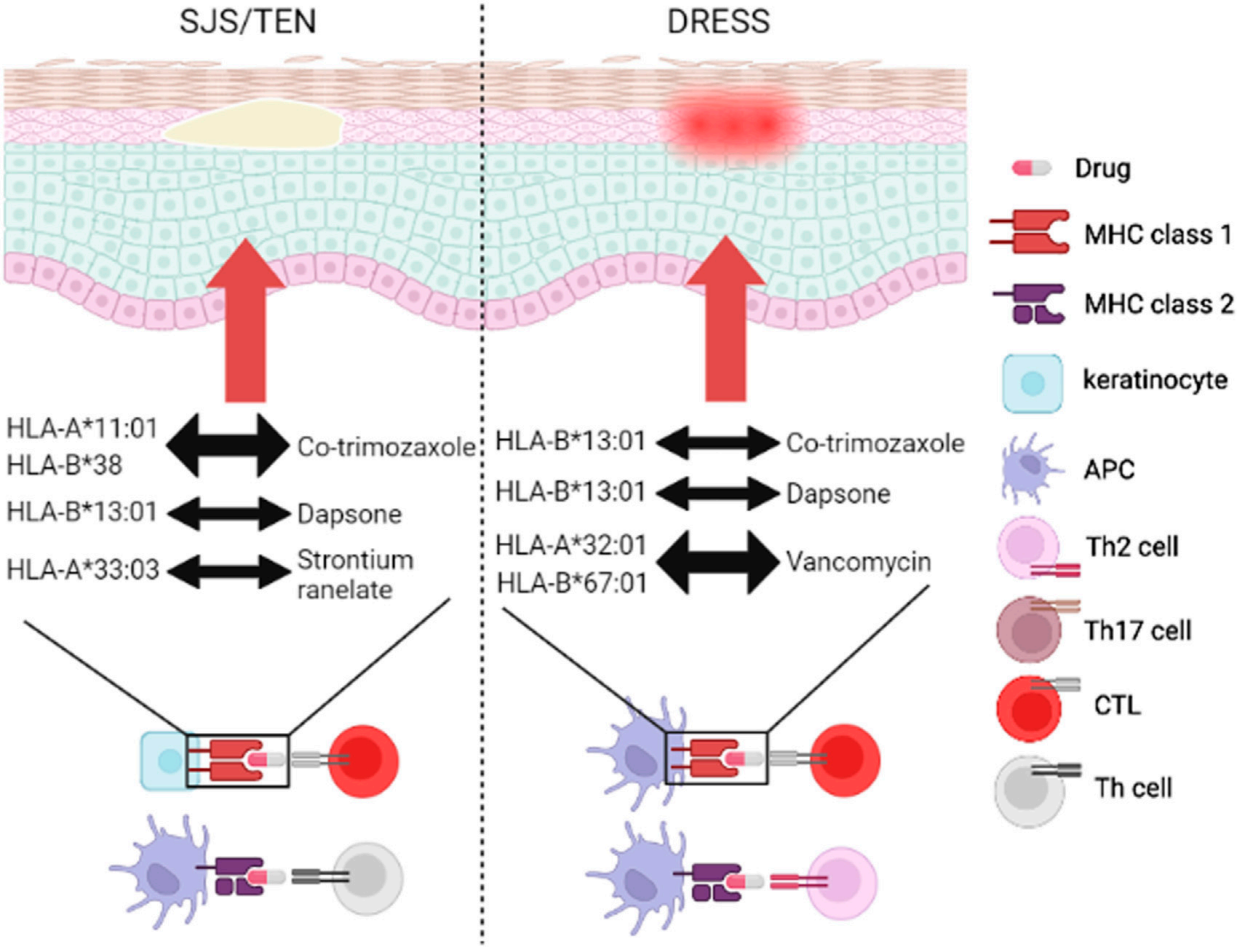
Genetic association of SCARs between HLA and drugs. The drugs (antibiotics or AODs) trigger DHRs through MHC/drug/TCR complex. SJS/TEN mainly 694 started immune mechanism by HLA/drug/CTLs and HLA/drug/Th cells, while DRESS mainly 695 started immune mechanism by HLA/drug/CTLs and HLA/drug/Th2 cells. Co-trimozaxole can induce 696 SJS/TEN and DRESS through binding to HLA-A*11:01/HLA-B*38 and HLA-B*13:01, 697 respectively. Dapsone can induce SJS/TEN and DRESS through binding to HLA-B*1301. 698 Vancomycin can induce DRESS by binding to HLA-A*32:01 and HLA-B*67:01. SR can activate 699 SJS/TEN through binding to HLA-A*33:03. Abbreviations: AOD, anti-osteoporotic drug; APC, antigen-presenting cell; CTL, cytotoxic T 701 lymphocyte; DHR, delayed hypersensitivity reaction; DRESS, drug reaction with eosinophilia and 702 systemic symptoms; HLA, human leukocyte antigen; MHC, major histocompatibility complex; SJS, 703 Stevens-Johnson syndrome; TCR, T cell receptor; TEN, toxic epidermal necrolysis; Th cell, T helper 704 cell.

Several antibiotics- and strontium ranelate (SR)-induced SCARs have been proposed in recent 2 decades (Blumenthal et al., 2019), and we have illustrated in Figure 1 (Administration, 2023). We summarize the relationships between well-known antibiotics and HLA-related ADRs listed in Table 1.

**TABLE 1:** Genetic associations with HLA in DHRs.

Causative drug	Genetic factor	Ethnicity	ADR	OR	p-value	Ref
Co-trimoxazole (Trimethoprim- sulfamethoxazole)	HLA- B*13:01	Chinese, Thai, Malaysian	DRESS	45 (18.7–134)	1.1 x 10^−26^	Wang et al. (2021)
			DRESS	3.88 (1.56–9.63)	0.0025	Sukasem et al. (2020)
		Thai	DRESS	8.44 (2.66–26.77)	2.94 x 10^−4^	Nakkam et al. (2022)
	HLA- B*15:02	Taiwan	SJS/TEN	2.7 (1.2-5.7)	0.008	Wang et al. (2021)
		Thai	SJS/TEN	3.47 (1.25–9.63)	0.0201	Sukasem et al. (2020)
			SJS/TEN	3.91 (1.42–10.92)	0.0037	Kongpan et al. (2015)
	HLA-C*06:02	Thai	SJS/TEN	11.84 (1.24–566.04)	0.0131	Kongpan et al. (2015)
	HLA-C*08:01	Thai	SJS/TEN	3.53 (1.21–10.40)	0.0108	Kongpan et al. (2015)
	HLA-B*38	European	SJS/TEN	8.6 (3.5–21)	< 10^−4^	Lonjou et al. (2008)
	HLA-B*38:01	European	SJS/TEN	4.3 (1.4–12.7)	0.022	Lonjou et al. (2008)
	HLA-B*38:02	Chinese,	SJS/TEN	2.5 (1.4–4.3)	0.003	Wang et al. (2021)
		Thai				
	HLA- A*11:01	Japanese	SJS/TEN	14.77 (2.97–73.4)	4.91 x 10^−4^	Nakamura et al. (2020)
			DRESS	6.56 (1.46–29.4)	0.0187	Nakamura et al. (2020)
Penicillins (Benzylpenicillin, Ampicillin, Amoxicillin, Cloxacillin)	HLA-B*55:01	European	Allergy	1.30 (1.25–1.34)	10^−47^	Krebs et al. (2020)
	HLA-B*48:01	Thai	Allergy	35.18 (1.64–753.16)	0.023	Singvijarn et al. (2021)
	HLA-C*04:06	Thai	Allergy	20.56 (1.78–237.92)	0.016	Singvijarn et al. (2021)
	HLA-C*08:01	Thai	Allergy	7.5 (1.92–29.36)	0.009	Singvijarn et al. (2021)
	HLA-	Italian	Allergy	8.9 (3.4–23.3)	< 0.001	Romano et al. (2022)
	DRB3*02:02					
Dapsone	HLA- B*13:01	Chinese, Thai	DRESS	49.64 (5.89–418.13)	2.92 x 10^−4^	Wang et al. (2013), Zhang et al. (2013), Chen et al. (2018b)
			DRESS	122.1 (23.5–636.2)	6.04 x 10^−^12	

			DRESS	40.50 (6.38-257.03)	2.37 x 10^−4^	
		Thai	SJS/TEN	36.00 (3.19–405.89)	0.0476	Satapornpong et al. (2021)
			SCAR	26.11 (7.27–93.75)	10^−^4	Satapornpong et al. (2021)
		Indonesian	Hypersensitivity	328.87 (1.44–106.7)	1.32 x 10^−7^	Krismawati et al. (2020)
	HLA-C*03:04	Thai	SCAR	9.00 (2.17–37.38)	0.0464	Satapornpong et al. (2021)
Vancomycin	HLA- A*32:01	European	DRESS	403 (20.69–7849.44)	10-8	Konvinse et al. (2019)
		Spanish	DRESS	6.36 (1.68–24.13)	0.014	Bellon et al. (2022)
		Chinese	DRESS	7.8 (1.7–35.8)	0.035	Wang et al. (2022c)
	HLA-B*07:05	Chinese	DRESS	32.3 (2.8–367.7)	0.047	Wang et al. (2022c)
	HLA-B*40:06	Chinese	DRESS	4.7 (1.3–16.1)	0.036	Wang et al. (2022c)
	HLA-B*67:01	Chinese	DRESS	44.8 (7.2–280.4)	0.002	Wang et al. (2022c)
Clindamycin	HLA-B*51:01	Chinese	cADR	9.73 (2.93–32.35)	0.0018	Yang et al. (2017)
	HLA-B*15:27	Chinese	cADR	55.6 (4.65–665.24)	0.0138	Yang et al. (2017)
Strontium ranelate	HLA- A*33:03	Chinese	SJS/TEN	19.4 (2.0–188.0)	0.006	Lee et al. (2016)
		Chinese	SJS	25.97 (3.08–219.33)	5.17 x 10^−3^	Chen et al. (2021b)
	HLA-B*58:01	Chinese	SJS/TEN	8.0 (1.2–53.1)	0.042	Lee et al. (2016)

Abbreviation: cADR, cutaneous adverse drug reaction; DRESS, drug reaction with eosinophilia and systemic symptoms; HLA, human leukocyte antigen; SCAR, severe cutaneous adverse reactions; SJS, Stevens-Johnson syndrome; TEN, toxic epidermal necrolysis.

### 3.1 Co-trimoxazole (Trimethoprim-sulfamethoxazole)

Co-trimoxazole is an antibiotics indicated for urinary tract infection and Pneumocystis jiroveci pneumonia (Haseeb et al., 2022; Jent et al., 2022). The prevalence of skin adverse reaction was 1%–4% among general population treated with co-trimoxazole (Wolkenstein et al., 1995; Masters et al., 2003). Lonjou et al. (2008) found association between HLA-B*38 and co-trimoxazole-induced SJS/TEN in European population, although ORs and statistical power showed borderline efficacy. On the contrary, a whole genome sequencing study revealed strong association between HLA-B*38:02 and co-trimoxazole-induced SJS/TEN in Chinese and Thai population. In addition, Wang et al. (2021) found association between HLA-B*15:02 and co-trimoxazole-induced SJS/TEN in Taiwanese and Thai population (Wang et al., 2021). In Thai population, HLA-C*06:02 and HLA-C*08:01 were also associated with co-trimoxazole-induced SJS/TEN (Kongpan et al., 2015). To our best knowledge, co-trimoxazole-induced DRESS has been reported to be related to HLA-B*13:01 among Chinese, Thai and Malaysian population and HLA-A*11:01 among Japanese population (Nakamura et al., 2020; Sukasem et al., 2020; Wang et al., 2021; Nakkam et al., 2022). Pratoomwun et al. (2021) found co-trimoxazole directly binding to HLA-B*13:01 induced drug-specific T cell response and further immune mechanism including IL-13, IFN-γ, granzyme B, and IL-22 secretion in Thai population.

### 3.2 Penicillins

Penicillin and its derivatives are antimicrobial agents frequently used to treat a variety of bacterial infection in the world (Chang et al., 2020). Zhou et al. (2016) reported that the prevalence of penicillin allergy was 12.8%. Penicillins may cause drug hypersensitivity-related skin lesions, varying from skin rash to SCARs (Lin et al., 2014). According to Krebs et al. (2020) genome-wide associated study (GWAS), HLA-B*55:01 is a genetic biomarker for penicillin allergy in European population. Another GWAS involving 5 countries (Australia, France, Italy, Spain, and United Kingdom) concluded that HLA-DRB*10:01 is associated with immediate penicillin hypersensitivity, but not delayed penicillin hypersensitivity (Nicoletti et al., 2021). Romano et al. (2022) conducted next-generation sequencing (NGS) and found that HLA-DRB3*02:02 is associated with delayed penicillin hypersensitivity in Italian population. A case-control study unveiled that HLA-C*04:06, HLA-C*08:01, and HLA-DRB1*04:06 are associated with β-lactam delayed reaction in Thai children (Singvijarn et al., 2021).

### 3.3 Piperacillin-Tazobactam

Piperacillin is an extended-spectrum penicillin usually found in combination with tazobactum, a β-lactamase inhibitor (Drawz and Bonomo, 2010). Piperacillin-tazobactam could cover most of the gram-positive and gram-negative bacteria, including *Pseudomonas aeruginosa* (Wong et al., 2021). To our best understanding, only one observation study revealed that HLA-B*62 might be associated with piperacillin-tazobactam induced DRESS in European population (Rutkowski et al., 2017). A larger sample size may be needed to validate the above association.

### 3.4 Dapsone

Dapsone, a sulfone drug with anti-microbial and anti-inflammatory effects, has been used for leprosy and dermatitis herpetiformis (Reunala et al., 2021; Li et al., 2022). The prevalence of dapsone-induced SCARs was 0.5%–3.6% after 4–6 weeks treatment (Rao and Lakshmi, 2001). According to Zhang et al. (2013) GWAS study, HLA-B*13:01 is associated with dapsone-induced hypersensitivity in Chinese population. The association between HLA*B13:01 and dapsone-induced DRESS was reported in Taiwan and Thai population, respectively (Wang et al., 2013; Chen et al., 2018b; Satapornpong et al., 2021). Krismawati et al. validated HLA-B*13:01 as biomarker of dapsone-induced hypersensitivity in leprosy patients among Indonesian population (Krismawati et al., 2020). Two meta-analysis demonstrated the association between HLA-B*13:01 and dapsone-induced SCARs in Chinese and Southeastern Asian population (Tangamornsuksan and Lohitnavy, 2018; Park et al., 2020). Most studies have revealed the relationship between HLA-B*13:01 and dapsone-induced DRESS (Jung et al., 2018). Satapornpong et al. found that HLA-C*03:04 is associated with dapsone-induced SCARs, although dapsone-induced DRESS and SJS/TEN showed no significance owing to weak statistical power (Satapornpong et al., 2021). In addition, Lee et al. (2022) analyzed the clinical data warehouse from Korea and found there is no association between HLA-B*13:01 and dapsone-induced SCARs in Korean population. Zhao et al. (2021) investigated that dapsone and its metabolite nitroso-dapsone are selectively interacted with HLA-B*13:01 to activate CTLs related cytotoxicity. Recently, Jiang et al. (2022) demonstrated that HLA-B*13:01-dapsone-TCR immune molecular mechanism is formed according to pharmacological-interaction model.

### 3.5 Vancomycin

Vancomycin, a glycosylated peptide antibiotic, has been used mainly for resistant gram-positive bacteria-related infection (Dinu et al., 2020). According to a recent large electronic healthcare record database, the prevalence of vancomycin-induced DRESS was 39% (Pirmohamed, 2019; Wolfson et al., 2019). Furthermore, the literature on vancomycin-induced SJS/TEN is rare (Minhas et al., 2016; De Luca et al., 2020). Young et al. (2014) reported that there is no association between HLA alleles and vancomycin-induced DRESS, probably due to only three patients. HLA-A*32:01 is associated with vancomycin-induced DRESS in European population (Konvinse et al., 2019). Bellon et al. (2022) also revealed the association between HLA-A*32:01 and vancomycin-induced DRESS in Spanish population. Recently, we reported the association between HLA-A*32:01, HLA-B*07:05, HLA-B:40:06, HLA-B*67:01 and vancomycin-induced DRESS in Taiwanese population (Wang et al., 2022c). However, Lee et al. (2022) reported that there is no association between HLA-A*32:01 and vancomycin-induced SCARs in Korean population according to clinical data from Seoul National University Hospital. Nakkam et al. (2021) elucidated the possibility of cross-reactivity between vancomycin, teicoplanin, and dalbavancin in HLA*A-32:01 patients with previous vancomycin-induced DRESS. Ogese et al. (2021) reported that the direct interaction of vancomycin-induced DRESS between vancomycin and HLA*A-32:01 and further high expression of CXCR3, CCR4, IL-13, and IFN-γ.

### 3.6 Clindamycin

Clindamycin, a macrolide antibiotic, used for several bacterial infection including atypical pneumonia, middle ear infection and endocarditis (Dashti et al., 2022). Yang et al. (2017) reported that there is an association between HLA-B*51:01 as well as HLA-B*15:27 and clindamycin-related CADRs in Chinese population. To our best knowledge, no studies have reported the association between HLA alleles and clindamycin-induced SCARs.

### 3.7 Strontium ranelate (SR)

SR is used for treatment of severe osteoporosis (Yang et al., 2014). Although there is a moderate risk for SR-induced DRESS in French population (Agier et al., 2016), no further studies have reported the association between HLA alleles and SR-induced DRESS (Adwan, 2017). Lee et al. (2016) reviewed several cases in Singapore and found the association between HLA-A*33:03 as well as HLA-B*58:01 and SR-induced SJS/TEN in Chinese population. Recently, Chen et al. (2021b) demonstrated that there is an association between HLA-A*33:03 and SR-induced SJS in Taiwanese population.

## 4 Application of HLA testing in clinical practice

Diagnosis of drug delayed hypersensitivity reaction is often tardy leading to multiple complications and even death (Kulkarni et al., 2022). Different population and ethnicity possess varied genetic HLA molecules, accountable for different degree of drug delayed hypersensitivity reactions (Jung et al., 2018). Besides, the frequency of HLA alleles and cost of prevention and management of ADR varied from one country to another, as well (Kloypan et al., 2021).

According to the Allele Frequency Net Database (AFND), the frequency of HLA-B*13:01 is 8%–15% in Taiwan and China. Co-trimoxazole-induced DRESS can be avoided after HLA-B*13:01 testing in Asian population. Although the frequency of HLA-A*11:01 allele is lower in Japanese population compared to Chinese and Thai population (Gonzalez-Galarza et al., 2018), HLA-A*11:01 is a high risk factor involved in co-trimoxazole-induced SJS/TEN in Japanese population (Nakamura et al., 2020). Even though lots of co-trimoxazole-induced DRESS have been reported, there is still no specific HLA alleles in European population. Larger scales of studies are required to identify the relevant HLA alleles in European population (Wang et al., 2021). Another example is dapsone and HLA-B*13:01. The frequency of HLA-B*13:01 is rare in European and African population, but occurs with frequency of 2%–20% in Chinese, 7% in Thai population and 1%–12% in Indian population (Puangpetch et al., 2014; Tempark et al., 2017). Moreover, HLA-B*13:01 testing is recommended for Chinese patients with leprosy before initiating dapsone therapy (Liu et al., 2019). Owing to the relatively high frequency of HLA-A*32:01 in European population, genetic examination before vancomycin treatment can be effective to prevent vancomycin-induced DRESS (Rwandamuriye et al., 2019; Bellon et al., 2022). Hama et al. (2022) reported that the frequency of HLA-A*32:01 is 6.8% in European population and 20% of whom developed vancomycin-induced DRESS. Unlike drug-induced SJS/TEN and DRESS, there has not been a definite link between drug-induced AGEP and specific HLA genotypes (Owen and Jones, 2021). Individuals with IL-36 RA deficiency seems to being subjected to drug-induced AGEP. However, it is still unclear how IL-36 RA deficiency leads to AGEP (Gabay and Towne, 2015).

## 5 Current trends and future perspectives

With the increasing number of published studies regarding genetic polymorphisms associated with drug delayed hypersensitivity reaction, HLA alleles of SCARs could be drug-specific, ethnicity-specific and phenotypic-specific (Tempark et al., 2022). Considering the morbidity rate, mortality rate and economic burden of SCARs, it is imperative for SCAR prevention to have an efficient and effective method (Yang et al., 2021). Serum granulysin level can be not only a potential marker for the early phase of SJS/TEN, but also a predictive marker for DRESS diagnosis and prognosis (Chung et al., 2008; Saito et al., 2012). Serum IL-15 level can become a marker for SJS/TEN early diagnosis and prognosis monitoring (Su et al., 2017). In addition, serum TARC level was identified as a potential biomarker for DRESS severity and prognosis (Komatsu-Fujii et al., 2018). Moreover, epigenetic association has been found in SCARs patients (such as ITGB2 methylation associated with allopurinol-induced SCARs) (Liu et al., 2023). Recently, the high-throughput technologies including whole genome sequencing (WGS) and whole exome sequencing (WES) offer us rapid method to screen the genetic variants (Wang et al., 2022a). A number of studies have advocated the use of pharmacogenetic testing in terms of cost-effectiveness. Compared to dealing with the life-threatening severe ADR, single HLA allele testing alleviates the cost. Currently, HLA-B*57:01 screening before abacavir treatment, HLA-B*15:02 screening before cabamazepine treatment and HLA-B*58:01 screening before allopurinol treatment have been a standard operation procedure for SCARs prevention (Hung et al., 2005; Mallal et al., 2008; Fan et al., 2017). Hopefully, with the increasingly studies regarding antibiotics as well as SR and HLA alleles, the promising HLA molecules will become standard screening before antibiotics prescription.
